# Crystal structure of vaccinia virus G3/L5 sub-complex reveals a novel fold with extended inter-molecule interactions conserved among orthopoxviruses

**DOI:** 10.1080/22221751.2022.2160661

**Published:** 2023-01-10

**Authors:** Sheng Lin, Dan Yue, Fanli Yang, Zimin Chen, Bin He, Yu Cao, Haohao Dong, Jian Li, Qi Zhao, Guangwen Lu

**Affiliations:** a West China Hospital Emergency Department (WCHED), State Key Laboratory of Biotherapy, West China Hospital, Sichuan University, Chengdu, People’s Republic of China; b Disaster Medicine Center, West China Hospital, Sichuan University, Chengdu, People’s Republic of China; c Laboratory of Aging Research and Cancer Drug Target, State Key Laboratory of Biotherapy and Cancer Center, National Clinical Research Center for Geriatrics, West China Hospital, Sichuan University, Chengdu, People’s Republic of China; d School of Basic Medical Sciences, Chengdu University, Chengdu, People’s Republic of China; e College of Food and Biological Engineering, Chengdu University, Chengdu, People’s Republic of China

Dear Editor,

Poxviruses from *Orthopoxvirus* genus of *Poxviridae* family are large, enveloped DNA viruses [1,2]. Of the 12 members within the species, some are important human viruses, e.g. monkeypox virus (MPXV, the recent monkeypox outbreak, as of 27 November 2022, has caused 81,107 infections and spread to 110 countries, territories or areas [3]), variola virus (VARV, the highly contagious and lethal pathogen that causes smallpox), vaccinia virus (VACV, a live, naturally attenuated vaccine used for smallpox and monkeypox prevention), etc. The continuous spread of orthopoxvirus has posed great threat to global public health. To combat the virus transmission, it is an urgent need to characterize the virus-encoded proteins associated with viral entry, thereby facilitating the development of more effective therapeutics.

Entry is the first step for a virus to initiate infection and also an important target for humoral immunity [4,5]. Unlike most other members of enveloped viruses that utilize one or a limited number of viral proteins for entry [6], poxvirus encodes four (A26, A27, D8 and H3, protein names refer to VACV) and another eleven proteins (A16, A21, A28, F9, G3, G9, H2, J5, L1, L5 and O3, protein names refer to VACV) to mediate attachment and membrane fusion, respectively [1,7]. For the eleven fusion associated proteins, they are proposed to assemble into a large machinery, called the entry-fusion complex (EFC) [1,7]. Individual genetic suppression of most VACV EFC constitutive proteins (A16, A21, A28, F9, G3, G9, H2, L1, L5 and O3) leads to the formation of mutant viruses with normal cell-binding capacity, but significantly reduced viral infectivity [8]. These results demonstrate that EFC proteins play an essential role in the post-attachment (hemifusion or full fusion) process of poxvirus life cycle [1,8]. Thus, structural studies on EFC components or complexes will help reveal the enigmatic fusion mechanism of EFC and further guide the development of prophylactic/therapeutic agents. However, until now, only two structures of the EFC components from VACV (F9 [9] and L1 [10]) have been experimentally determined.

In this study, we carried out structural characterization on VACV G3 and L5 proteins. These two EFC-component proteins, have been previously shown to interact with each other to form a stable sub-complex [11,12], and show highly conserved amino-acid sequences among orthopoxviruses (Figure S1A, B). The ectodomain of G3 consists of residues Y22-K111, and the ectodomain of L5 spans from N52 to R128 (Figure 1(A)). To verify the ectodomain binding between the two proteins, MBP/His-tagged G3-ectodomain (G3-Ecto) and MBP/FLAG-tagged L5-ectodomain (L5-Ecto) were co-expressed in *Escherichia coli* and then enzymatically cleaved to remove MBP (Figure 1(B)). Ion-exchange chromatography and western blot (anti His and FLAG) analysis showed that their ectodomain proteins formed sub-complex in solution and could be co-purified (Figure 1(C)). Subsequently, we prepared homogeneous tag-free protein of the G3-Ecto/L5-Ecto complex for crystallographic investigations (Figure 1(D,E)). Diffractable crystals for selenomethionine (SeMet)-substituted G3-Ecto/L5-Ecto complex were successfully obtained in conditions with two different space groups. Eventually, we solved the sub-complex structures at 1.8-Å (for *P*2_1_ space group) and 1.5-Å (for *P*3_1_ space group) resolutions using the single-wavelength anomalous dispersion method, respectively. The *R*_work_/*R*_free_ values were refined to 0.202/0.221 (for *P*2_1_ space group) and 0.190/0.206 (for *P*3_1_ space group), respectively (Table S1). Because protein terminus residues are much better traced in the electron density map for the structure with the *P*2_1_ space group than the structure with the *P*3_1_ space group (Figure S2A, B), description of the structural elements below is based on the *P*2_1_ structure unless otherwise specified.

**Figure 1.**
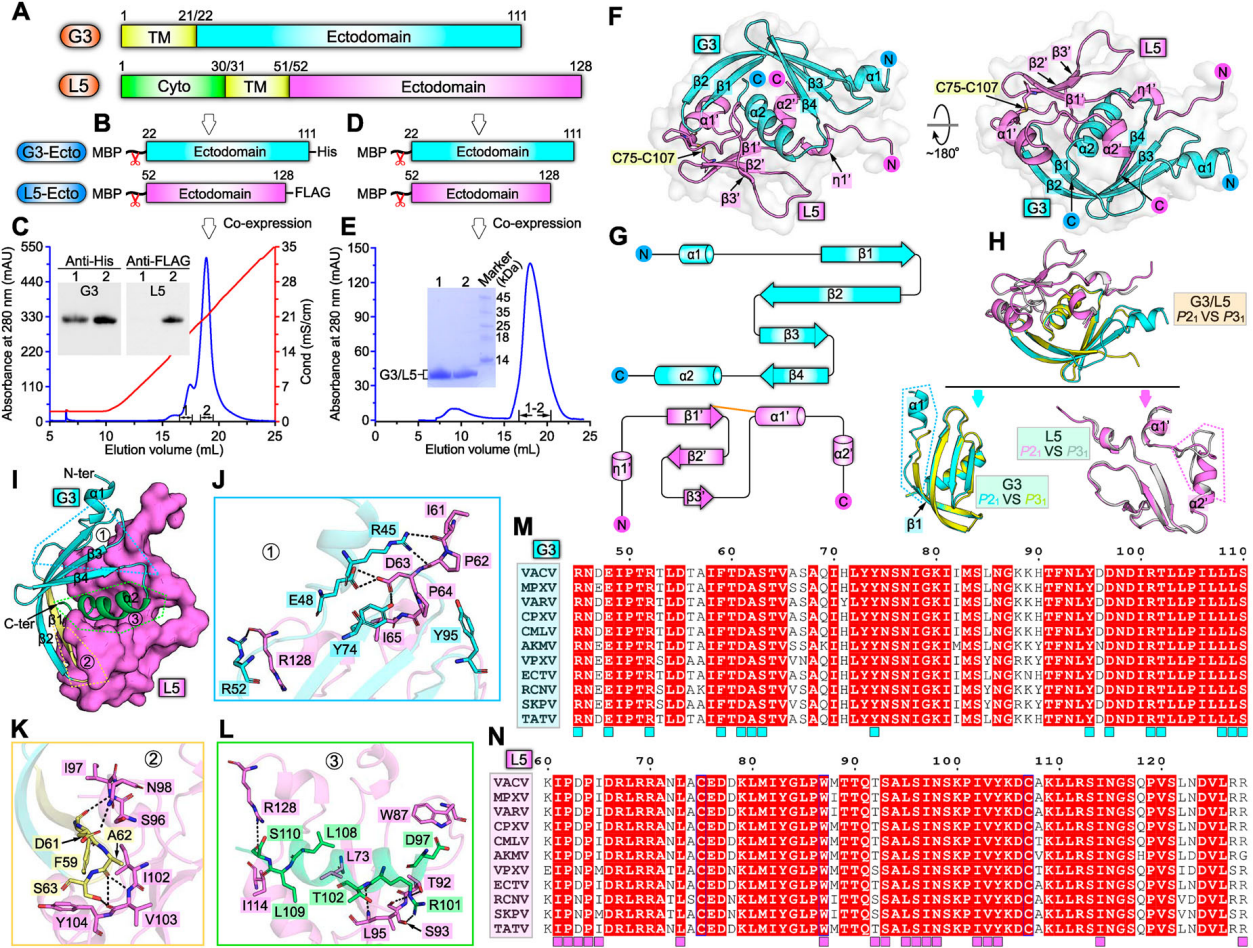
Structure of VACV G3/L5 sub-complex. A schematic view of the protein-engineering strategy used to yield VACV G3-Ecto-His/L5-Ecto-FLAG sub-complex (A, B) or tag-free VACV G3-Ecto/L5-Ecto sub-complex (A, D). The transmembrane domain (TM), the ectodomain, and the cytoplasmic domain (Cyto) are individually marked with the boundary-residue numbers. (C) Identification of G3-Ecto-His/L5-Ecto-FLAG hetero-complex using ion-exchange chromatography and western blot assay. (E) Solution behaviour of G3-Ecto/L5-Ecto sub-complex on a Superdex 200 Increase 10/300 GL column. (F) Overall structure of the heterodimer (*P*2_1_ space group) formed between G3 (cyan) and L5 (violet). (G) Topology plots of G3 and L5 proteins. (H) Structural comparison of G3/L5 complexes from two space groups (*P*2_1_ and *P*3_1_). Those elements exhibiting variant conformations are highlighted by dotted box. (I-L) The atomic binding details between G3 and L5. Residues providing ≥10 contacts are shown and labelled. Dashed lines indicate hydrogen bonds. (M) Multiple sequence alignment of the G3 homologues from orthopoxviruses. Key residues on VACV G3 that interact with L5 are marked with cyan squares. (N) Multiple sequence alignment of the L5 homologues from orthopoxviruses. Key residues on VACV L5 that interact with G3 are marked with violet squares.

The complex structure, within the crystallographic asymmetric unit, contains G3 (amino acids A40-K111) and L5 (residues K60-R128) in single 1:1 binding mode. As can be viewed from the overall structure, G3 and L5, with good shape complementarity, are stacked together to form a globular fold (Figure S3A, B). G3 consists of four anti-parallel β-strands (β1-β4) and two α-helices (α1-α2), assembling into a compact structure (Figure 1(F,G)). Spatially, the curving β-strands fold into a centrally-located β-sheet with a concave plane, cradling helix-α2 in the middle. Helix-α1 intersperses the β-sheet on the lateral side. L5 is composed of three anti-parallel β-strands (β1’-β3’), two α-helices (α1’-α2’) and one 3_10_ helix (η1’) (Figure 1(F,G)). One conserved disulfide bond is observed between C75 in β1’-strand and C107 in α1’-helix to stabilize the structure (Figure 1(F); Figure S1B). Structural homology searches against whole PDB database using DALI server [13], however, did not yield significant match to either G3 or L5 structure, indicating that these two proteins both adopt a novel fold.

As expected, the structure in the *P*3_1_ space group also comprises a 1:1 G3/L5 heterodimer with the spatial arrangement and binding pattern of each protein the same as those in the *P*2_1_ structure (Figure 1(H)). Nevertheless, apparent conformational differences are observed in (1) the N-terminal α1-helix is completely untraceable in the *P*3_1_-G3 structure and (2) a portion of α2’-helix unfolds into a loop and partial of the α1’/α2’ intervening loop adopts a significantly variant conformation in the *P*3_1_-L5 structure (Figure 1(H)). Since the G3/L5 sub-complex, along with other EFC component proteins, can further form higher-order complex(es). The two conformationally-alterable parts observed in our two pairs of heterodimeric structures may play a role in interacting with other components for EFC assembly.

Guided by the high-resolution complex structure, we then characterized the atomic binding details between G3 and L5. On the whole, large surface areas are buried in the sub-complex, which are calculated to be ∼1606 Å^2^ for G3 and ∼1553 Å^2^ for L5, respectively. The extended binding interface can be further subdivided into three binding patches (Patch1, 2 and 3) based on the G3 elements involved in L5-engagement. Patch1 mainly involves helix-α1, strand-β2 and the α1/β1 and β4/α2 intervening loops of G3 (Figure 1(I)), forming an extensive interaction network with L5. Located within this patch include residues of G3 protein R45, E48, R52, Y74 and Y95 and amino acids of L5 protein I61, P62, D63, P64, I65 and R128. A total of six inter-molecule hydrogen bonds (G3 protein-R45 with L5 protein-I61, R45 with P62, E48 with D63, R52 with R128, and Y74 with D63) are observed to form (Figure 1(J)). Patch2 mainly involves strand-β1 and β1/β2 intervening loop of G3 (Figure 1(I)). Residues F59, D61, A62 and S63 from the G3 protein and amino acids S96, I97, N98, I102, V103 and Y104 from the L5 protein are converged together to form multiple van der Waals and hydrogen-bond (G3 protein-F59 with L5 protein-I97, D61 with N98, A62 with V103, S63 with V103) interactions (Figure 1(K)). Patch3 mainly involves helix-α2 and C-terminal loop of G3 (Figure 1(I)). This patch features with a largest number of amino acids for inter-molecule contacts, including the G3 residues D97, R101, T102, L108, L109 and S110 and the L5 residues L73, W87, T92, S93, L95, I114 and R128. Of these amino acids, nearly half are of apolar side-chains, suggesting that a considerable part of the binding is mediated by hydrophobic interactions. In addition, five additional hydrogen bonds (G3-protein R101 with L5-protein T92, R101 with S93, R101 with L95, S110 with R128) are observed to form to further stabilize the engagement (Figure 1(L)). In comparison to the other two patches, the third patch therefore shows the best overall structural complementarity, the largest number of participating amino acids, and the most diverse types of inter-chain contacts (van der Waals, hydrophobic interactions and hydrogen bonds). Taken together, a total of 15 residues in G3 and 18 amino acids in L5 are positioned along the binding interface, providing extended inter-molecule interactions for sub-complex formation (Figure 1(M,N)). It is also worth noting that these key residues identified in the VACV G3/L5 binding interface are highly conserved among orthopoxviruses, including the monkeypox virus (Figure 1(M,N)). These results imply that the orthopoxviruses would share quite similar binding patterns and likely the same interaction details upon “G3/L5” sub-complex formation.

Previous studies have demonstrated that VACV A26 protein (an acid-sensitive fusion suppressor which regulates mature virus membrane fusion in endocytic pathway) could interact directly with A16 and G9 but not with G3 and L5 proteins [14,15]. Thus, it is likely that G3/L5 functions in a similar way when poxvirus enters at the cellular membrane or via the endocytosis, making the sub-complex a potential target for antiviral development regardless of the virus entry-route. Since G3/L5 is shown to be critical for viral fusion [1,8], interfering with the interaction between G3 and L5 is expected to disrupt the integrity of EFC, thereby reducing or blocking viral entry. It is notable that the completely conserved α2 helix in G3 protein is highly reminiscent of the newly-identified stem-helix epitope in spike protein of severe acute respiratory syndrome coronavirus 2 [16]. In addition, we also identified several pockets along the binding interface between G3 and L5, which might be targeted by small-molecule inhibitors (Figure S4A, B). Therefore, we believe that the characterized G3/L5 interfacing elements deserve further evaluation in vaccine and drug development to block the subunit engagement.

In conclusion, our high-resolution structures of the VACV G3/L5 sub-complex provide, to our knowledge, first insights into their component folding and interaction patterns. These data will not only facilitate the development of broad-spectrum agents targeting the conserved complex, but also serve as a new starting point to explore the assembly of higher-order complex(es) of EFC.

## Supplementary Material

Supplemental Material

## Data Availability

The datasets used and/or analysed during this study are available from the corresponding author on reasonable request. Atomic coordinates and structure factors for the reported crystal structures have been deposited into the Protein Data Bank under accession numbers 7YTT and 7YTU.
