# Supplementary material for: Crystal structure of vaccinia virus G3/L5 sub-complex reveals a novel fold with extended inter-molecule interactions conserved among orthopoxviruses
Source: Emerg Microbes Infect. 2023 Jan 10;12(1):e2160661. doi: 10.1080/22221751.2022.2160661 (PMC9848366; doi:10.1080/22221751.2022.2160661)
Supplement: Supplemental Material [file TEMI_A_2160661_SM9795.docx]

Supplementary materials for

**Crystal structure of vaccinia virus G3/L5 sub-complex reveals a novel fold with extended inter-molecule interactions conserved among orthopoxviruses**

Sheng Lin^1#^, Dan Yue^1#^, Fanli Yang^1^, Zimin Chen^1^, Bin He^1^, Yu Cao^1,2^, Haohao Dong^3^, Jian Li^4^, Qi Zhao^5^, Guangwen Lu^1*^

^1^ West China Hospital Emergency Department (WCHED), State Key Laboratory of Biotherapy, West China Hospital, Sichuan University, Chengdu, Sichuan 610041, China

^2^ Disaster Medicine Center, West China Hospital, Sichuan University, Chengdu, Sichuan 610041, China

^3^ Laboratory of Aging Research and Cancer Drug Target, State Key Laboratory of Biotherapy and Cancer Center, National Clinical Research Center for Geriatrics, West China Hospital, Sichuan University, Chengdu, Sichuan 610041, China

^4^ School of Basic Medical Sciences, Chengdu University, Chengdu, Sichuan 610106, China

^5^ College of Food and Biological Engineering, Chengdu University, Chengdu, Sichuan 610106, China

**# These authors contributed equally:** Sheng Lin, Dan Yue

*** Correspondence:** Guangwen Lu ([lugw@scu.edu.cn](mailto:lugw@scu.edu.cn))

**Materials and methods**

**Plasmid construction**

Two kinds of plasmid pairs for Vaccinia virus (Western Reserve strain) G3/L5 sub-complex were constructed, with one for complex identification and another for crystallization screening. For plasmids used for the subsequent complex-binding verification, the coding sequences for the ectodomain of G3 (GenBank: AAO89358.1, amino acids Y22-K111, with a C-terminal His-tag) and L5 (GenBank: AAO89371.1, residues N52-R128, with a C-terminal FLAG-tag) were cloned into pET-30a-MBP (modified pET-30a vector that contains an N-terminal MBP tag) and pET-21a-MBP (modified pET-21a vector that contains an N-terminal MBP tag), respectively. For plasmids used for the subsequent crystallization screening, the plasmids constructions were the same as above, but the extra His-tag on G3 and FLAG-tag on L5 were removed.

**Expression and purification of G3/L5 sub-complex**

For recombinant protein production, pair-wise plasmids were co-transformed into *Escherichia coli* BL21 (DE3) and expressed when 0.5 mM isopropyl-β-D-thiogalactoside (IPTG) was added at OD_600_=0.8. After induction at 16℃ for 16 h, the cells were harvested, resuspended with 20 mM Tris-HCl (pH 8.0) and 500 mM NaCl, lysed by sonication and centrifuged at 18,000 g for 30 min at 4℃. For preliminary purification, proteins were passed over gravity column loaded with Dextrin-Sepharose resin (GE Healthcare), washed with the buffer containing 20 mM Tris-HCl (pH 8.0) and 500 mM NaCl to remove contaminated proteins, and then eluted with the aforementioned buffer including 20 mM maltose. The targeted proteins were digested by PreScission Protease (PSP), then further purified using protein purification system (SDL-030-F2, Sepure Instruments Inc.) by ion-exchange chromatography (Source 15S column, GE Healthcare) and the next gel-filtration chromatography (Superdex 200 Increase 10/300 GL column, GE Healthcare). The selenomethionine (SeMet)-substituted G3/L5 sub-complex was expressed in M9 medium supplemented with Se-Met and purified similarly to the native protein.

**Western blot assay**

The protein fractions (containing G3-His or L5-FLAG) that collected from ion-exchange chromatography were directly subjected to western blot identification. The proteins were loaded and separated on an SDS-PAGE gel and transferred to polyvinylidene fluoride (PVDF) membranes. The membranes were blocked with 5% non-fat milk in Tris-buffered saline containing 0.1% Tween-20 (TBS-T) for 2 h at room temperature, and then incubated with primary antibodies against His-tag (1:2000 dilution) or FLAG-tag (1:2000 dilution) overnight at 4°C. Subsequently, the membranes were incubated with horseradish peroxidase-conjugated secondary antibodies at 37°C for 1 h. The bands were finally detected using the enhanced chemiluminescence system.

**Crystallization**

Commercial crystallization kits (Molecular Dimensions and Hampton Research) were used for initial crystallization screenings by the vapour-diffusion sitting-drop method. In brief, 1 µL tag-free native or SeMet-substituted G3/L5 protein was mixed with 1 µL reservoir solution, and the resultant mixture was then equilibrated against 70 µL reservoir solution at 18°C. Diffractable crystals for SeMet-labeled sub-complex (with protein concentration of 10 mg/mL) were successfully obtained in the following conditions with two different space groups. Condition 1 (*P*2_1_ space group): 0.2 M Ammonium nitrate (pH 6.2) and 20% w/v Polyethylene glycol 3,350. Condition 2 (*P*3_1_ space group): 0.1 M Sodium formate (pH 7.0) and 12% w/v Polyethylene glycol 3,350.

**Data collection and structure determination**

For data collection, crystals were flash-cooled in liquid nitrogen after a brief soaking in reservoir solution supplemented with 20% (v/v) glycerol. Diffraction data were collected at Shanghai Synchrotron Radiation Facility (SSRF) beamline BL19U1 [1]. The collected data were processed with HKL2000 [2] for indexing, integration and scaling. The initial phases and models of G3/L5 sub-complexes were determined by single-wavelength anomalous dispersion (SAD) method using the SHELXC/D/E program [3]. Then, the models were further rebuilt and adjusted manually with Coot [4], and refined by phenix.refine in PHENIX [5]. The ﬁnal data processing and structure reﬁnement statistics are summarized in Table S1. All structural ﬁgures were generated using PyMOL (<https://pymol.org/>).

**Sequences used in this study**

The GenBank accession numbers of the sequences (G3 and L5 homologues) used for structure-based multiple sequence alignment are as follows: VACV (vaccinia virus), GenBank: AAO89358.1 and AAO89371.1; MPXV (monkeypox virus), GenBank: URK20508.1 and URK20521.1; VARV (variola virus), GenBank: AAA60812.1 and AAA60825.1; CPXV (cowpox virus), GenBank: ADZ30272.1 and ADZ30285.1; CMLV (camelpox virus), GenBank: AAL73785.1 and AAL73797.1; AKMV (akhmeta virus), GenBank: AXN74871.1 and AXN74885.1; VPXV (volepox virus), GenBank: AOP31768.1 and AOP31781.1; ECTV (ectromelia virus), GenBank: AAM92367.1 and AAM92380.1; RCNV (raccoonpox virus), GenBank: AKJ93708.1 and AKJ93721.1; SKPV (skunkpox virus), GenBank: AOP31557.1 and AOP31570.1; TATV (taterapox virus), GenBank: ABD97648.1 and ABD97661.1.

**References**

1. Zhang W-Z, Tang J-C, Wang S-S, et al. The protein complex crystallography beamline (BL19U1) at the Shanghai Synchrotron Radiation Facility. Nuclear Science and Techniques. 2019;30(11):170.

2. Otwinowski Z, Minor W. Processing of X-ray diffraction data collected in oscillation mode. Methods Enzymol. 1997;276:307-26.

3. Schneider TR, Sheldrick GM. Substructure solution with SHELXD. Acta Crystallogr D Biol Crystallogr. 2002 Oct;58(Pt 10 Pt 2):1772-9.

4. Emsley P, Cowtan K. Coot: model-building tools for molecular graphics. Acta Crystallogr D Biol Crystallogr. 2004 Dec;60(Pt 12 Pt 1):2126-32.

5. Adams PD, Afonine PV, Bunkoczi G, et al. PHENIX: a comprehensive Python-based system for macromolecular structure solution. Acta Crystallogr D Biol Crystallogr. 2010 Feb;66(Pt 2):213-21.

**Supplementary Figures**


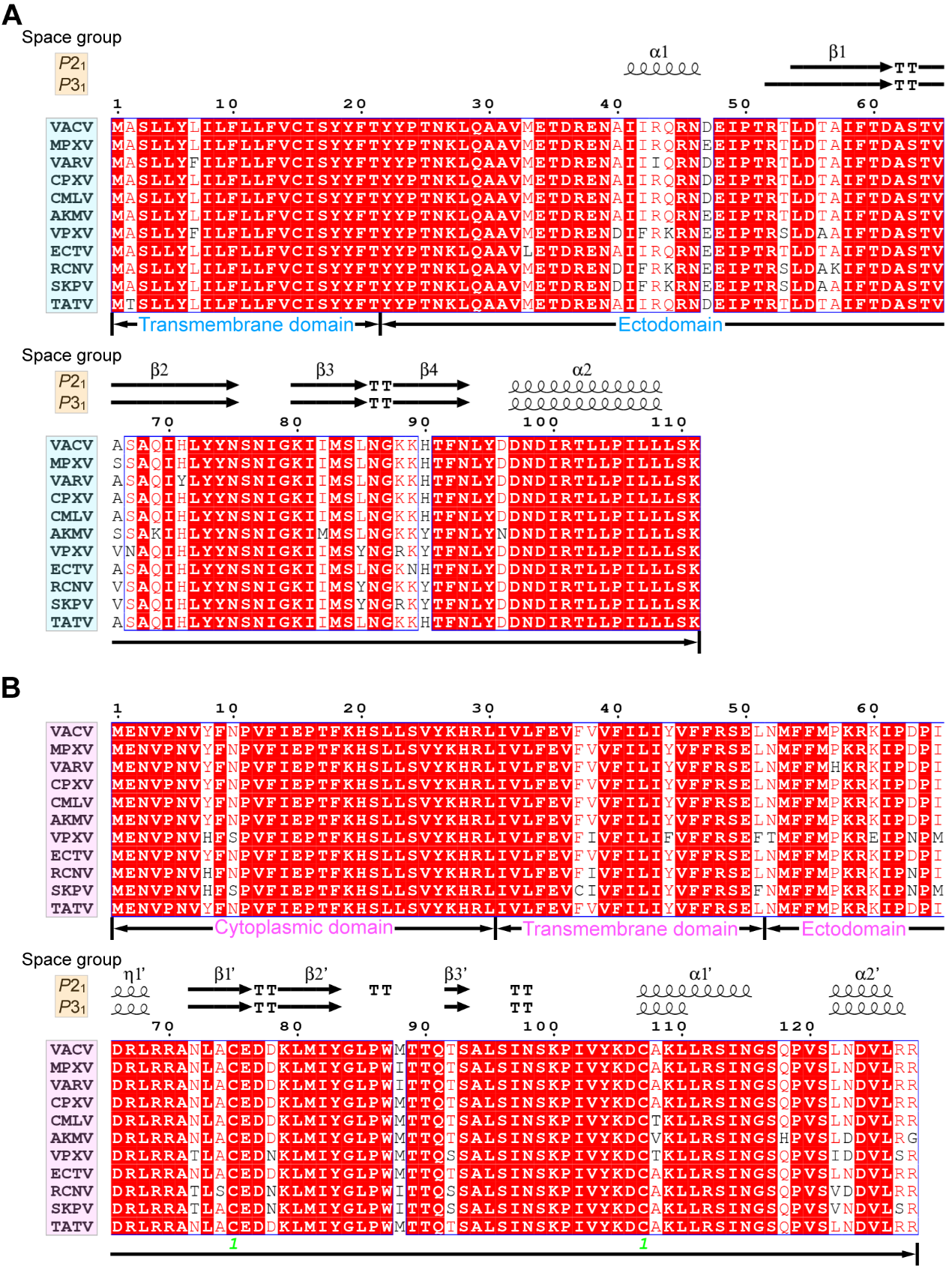


**Figure S1.** Structure-based multiple sequence alignment of the G3 and L5 homologues from representative orthopoxviruses. **(A)** Multiple sequence alignment for G3 homologues. **(B)** Multiple sequence alignment for L5 homologues. The secondary structural elements are labeled above the sequences, with horizontal arrows indicating β-strands and spinal lines representing α-helices or 3_10_ helices. The transmembrane domain, the ectodomain, and the cytoplasmic domain are marked below the sequences. The disulfide-related cysteine residues are labeled with italic number in green. Abbreviations: VACV (vaccinia virus), MPXV (monkeypox virus), VARV (variola virus), CPXV (cowpox virus), CMLV (camelpox virus), AKMV (akhmeta virus), VPXV (volepox virus), ECTV (ectromelia virus), RCNV (raccoonpox virus), SKPV (skunkpox virus), TATV (taterapox virus).


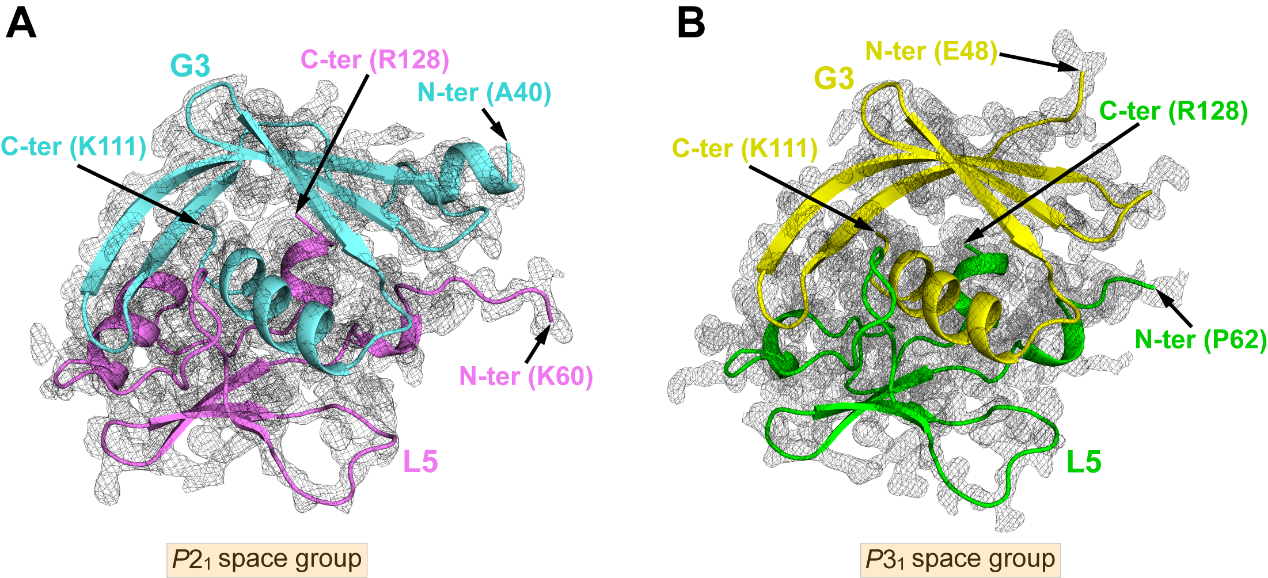


**Figure S2.** Overview of the electron density maps for the G3/L5 sub-complexes. **(A)** Electron density map for G3/L5 sub-complex in *P*2_1_ space group. **(B)** Electron density map for G3/L5 sub-complex in *P*3_1_ space group. The densities are depicted by contouring at 1.0 σ using the 2｜Fo｜-｜Fc｜map. The terminal residues that are density-traceable are labeled.


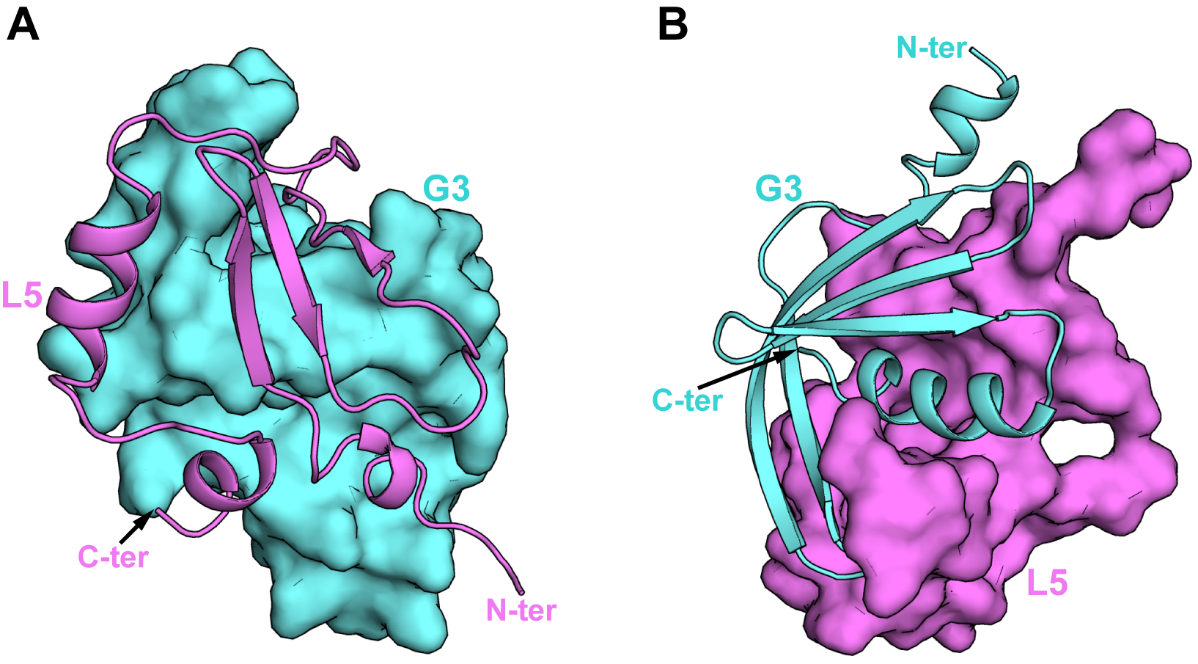


**Figure S3.** Shape complementarity between G3 and L5 upon complex formation. **(A)** G3 and L5 are shown in surface and cartoon representations, respectively. **(B)** G3 and L5 are shown in cartoon and surface representations, respectively.


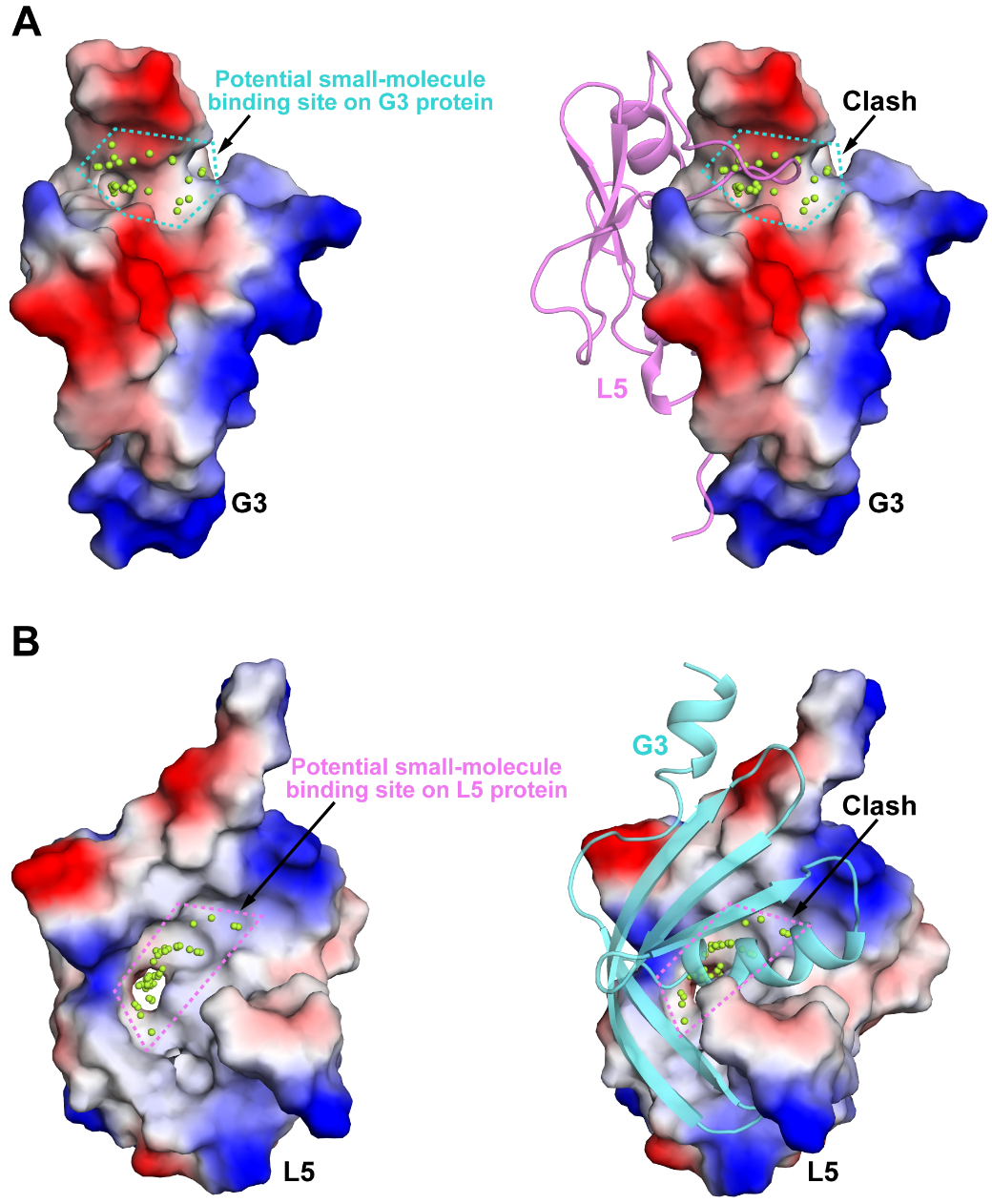


**Figure S4.** Exploration of potential small-molecule binding sites in G3 **(A)** and L5 **(B)** along their binding interface. The site ﬁnder function of the program Molecular Operating Environment (MOE) 2019.0102 (https://www.chemcomp.com/) was used to identify the targetable pockets, which are encircled with dashed lines and marked.

**Table S1.** Data collection and structure refinement statistics.

|  | SeMet-VACV G3/L5 (Condition 1) | SeMet-VACV G3/L5 (Condition 2) |
| --- | --- | --- |
| **Data collection** |  |  |
| Space group | *P*2_1_ | *P*3_1_ |
| Cell dimensions |  |  |
| *a*, *b*, *c* (Å) | 29.28, 41.92, 53.18 | 54.04, 54.04, 42.74 |
| *α*, *β*, *γ* (°)  Wavelength (Å) | 90, 92.54, 90  0.97892 | 90, 90, 120  0.97892 |
| Resolution (Å) | 50.00-1.80 (1.86-1.80) | 50.00-1.50 (1.55-1.50) |
| Unique reﬂections | 11,689 (1,143) | 22,077 (2,153) |
| *R*_merge_ | 0.113 (0.544) | 0.109 (0.611) |
| *I*/sig*I* | 15.87 (2.00) | 19.46 (2.43) |
| Completeness (%) | 99.4 (98.8) | 99.6 (96.7) |
| Redundancy | 5.2 (4.8) | 7.5 (6.5) |
|  |  |  |
| **Refinement** |  |  |
| Resolution (Å) | 29.25-1.81 | 31.56-1.50 |
| No. reflections | 11,674 | 22,050 |
| *R*_work_/*R*_free_ | 0.202/0.221 | 0.190/0.206 |
| No. of atoms |  |  |
| Protein | 1,119 | 1,034 |
| Ligand/ion | 0 | 0 |
| Water | 62 | 115 |
| *B*-factors (Å^2^) |  |  |
| Protein | 39.7 | 28.7 |
| Ligand/ion | - | - |
| Water | 43.2 | 38.7 |
| R.m.s. deviations |  |  |
| Bond lengths (Å) | 0.005 | 0.006 |
| Bond angles (°) | 0.772 | 0.955 |
| Ramachandran plot (%)  Favored region  Allowed region  Outlier region | 97.08  2.92  0 | 96.06  3.94  0 |
| **PDB code** | 7YTT | 7YTU |

A single crystal was used to collect the data.

Values in parentheses are for the highest-resolution shell.
